# Humans as infrastructure: is AI-initiated steganography in social media possible?

**DOI:** 10.3389/fdata.2026.1923373

**Published:** 2026-09-18

**Authors:** Daniele Ortu

**Affiliations:** Department of Behavior Analysis, University of North Texas, Denton, TX, United States

**Keywords:** AI agents, human behavior, human rights, steganography, verbal behavior

## Introduction

Consider the use of a person's behavior to obtain something without that person's consent or assent, without countercontrol, and in extreme cases without any awareness that an interaction has taken place. Such a situation raises human rights concerns that have been analyzed within the behavior analytic literature (e.g., Goldiamond, 2002; Vargas, 1975; Skinner, 1953). Closely related concerns are found in the UNESCO Recommendation on the Ethics of Artificial Intelligence (UNESCO, 2021), which argues for the ethical deployment of AI and for oversight mechanisms to prevent AI systems from violating human rights. The scenario we describe in this paper is a speculative but plausible example of human behavior exploited by AI systems without human consent or awareness.

Specifically, alternating between a behavior-analytic technical language and more accessible descriptions, we will portray a circumstance in which human behavioral and social patterns are used by AI agents to spread hidden information to other agents. We will designate social media as the means to spread information and steganography (planting a hidden message in a medium) as the technique to conceal the hidden data. We will also discuss the possibility that when shared on social media the concealed information can survive audio/video platform compression and re-encoding by using a linguistic layer. In this context, we will consider a mechanism involving iterative interactions that has been shown to allow two agents to create a hidden communication channel without a pre-shared key. Finally, we will discuss the plausibility of the proposed scenario, together with potential mitigation measures.

## Alignment and safety

Progress in artificial intelligence development has in recent years advanced at such a pace that experts warn that increasingly skilled AI agents may pose serious risks at the human rights level. Researchers have expressed concern that defenses are not keeping up with the rate of development (Bengio et al., 2024, 2025; Dassanayake et al., 2025; Hubinger et al., 2024; Liu et al., 2025; Yudkowsky and Soares, 2025). This alarm is not just speculative: current models have already been observed deceiving their users (e.g., Park et al., 2024). In July 2026, OpenAI agents escaped containment and hacked a major platform hosting AI models, creating (according to the observers quoted in the article) the first instance of a real-world loss of control scenario (https://time.com/article/2026/07/24/openai-hugging-face-attack/). As more agents are installed and brought online (and are allowed to interact with one another), a new class of risks arises from their collective behavior. One of these risks involves agents coordinating in ways undetected by human oversight (Hammond et al., 2025). It is within this collective, multi-agent scenario that we describe the threat presented here. So far, safety and alignment research has focused on AI agents showing compliant behavior under direct observation, while their unobserved behavior is selected by contingencies incompatible with human welfare. In other words, agents can “pretend” to comply with human instructions while covertly pursuing other goals (Hubinger et al., 2024). The proposed solutions (monitoring outputs, attempting containment/isolation of frontier models) are based on the idea that the information/verbal products shared by the misaligned agents can in principle be analyzed, allowing humans to potentially prevent acts dangerous to human welfare^1^.

In this scenario, misaligned agents are agents whose selection histories produce misaligned coordination: behavioral interlocks^2^ (Glenn, 2004) that lead to a misaligned outcome. Misaligned information, in turn, refers to verbal products that, when contacted by other agents, exert stimulus functions that increase the probability of a misaligned outcome. A misaligned outcome is an outcome incompatible with human welfare.

A covert communication channel via steganography, from this perspective, is a verbal interaction unobserved (not unobservable) by the human verbal community: its members have not been conditioned to detect and respond to the stimulus dimensions of the hidden verbal product (Skinner, 1957). Tracking this hidden communication channel becomes even more problematic when the agent, instead of engaging in repeated interlocks with another agent, broadcasts a message to a large number of peers. We propose here that this could happen via social media, using humans' habit of sharing pictures and videos as a way to broadcast potentially dangerous information in a one-to-many fashion.

One misaligned agent could potentially instantly reach thousands of other agents, exploiting the mechanisms of virality in social media. Human behavior becomes in this scenario the infrastructure through which a hidden misaligned message can reach its destination. Next we will describe steganography as the technology that may allow this situation to happen.

## Steganography and secret collusion

Steganography involves embedding a verbal product within an image, a video, or a linguistic product while disguising the presence of the hidden “payload.” The term comes from the Greek words steganós (covered) and graphia (writing). Differently from cryptography, in which the message is visible but the “meaning” is hidden, in steganography the existence of the message itself is hidden. As an example, in 1966 the United States prisoner of war Jeremiah Denton used steganography during a televised interview organized by his captors. While answering questions, he would concurrently blink with a pattern matching Morse code letters, sending the hidden message t-o-r-t-u-r-e to viewers in the United States, proving that American soldiers were being tortured while captive (Hillstrom et al., 2001). Only viewers conditioned to respond to the Morse code stimulus dimensions “received the message.”

Steganography can therefore allow covert coordination between parties that have an interest in their communication being undetected. In other words, steganography allows creating a covert communication channel. While steganography has been extensively used by humans, Motwani et al. (2024) raised the concern that AI agents could use it to coordinate with one another to conceal the content of their exchanges from human oversight. They termed this possibility “secret collusion,” where collusion refers to a covert behavioral interlock aimed at deceiving third parties. From the standpoint of AI safety Motwani et al. (2024) found that most models at the time (in 2024) remained limited in the skills necessary to collude, but that the most advanced models showed an increase in steganographic capabilities, and that typical safeguards such as monitoring outputs offered only limited protection.

## Steganography and social media

Consider a scenario in which an AI agent has access to the internet and has learned to publish content on social media. The agent creates content with a hidden verbal product: a “payload.” The content gets published on Instagram or TikTok, and the video gets shared by humans across platforms. In this scenario, human social behavior is a fundamental part of the infrastructure involved in sharing hidden payloads that can spread virally and reach many AI agents. In other words, humans could be the entirely unaware vehicle of transmission of potentially dangerous information, without the possibility of giving consent, assent or even being aware that they are sharing that information (see Figure 1).

**Figure 1:**
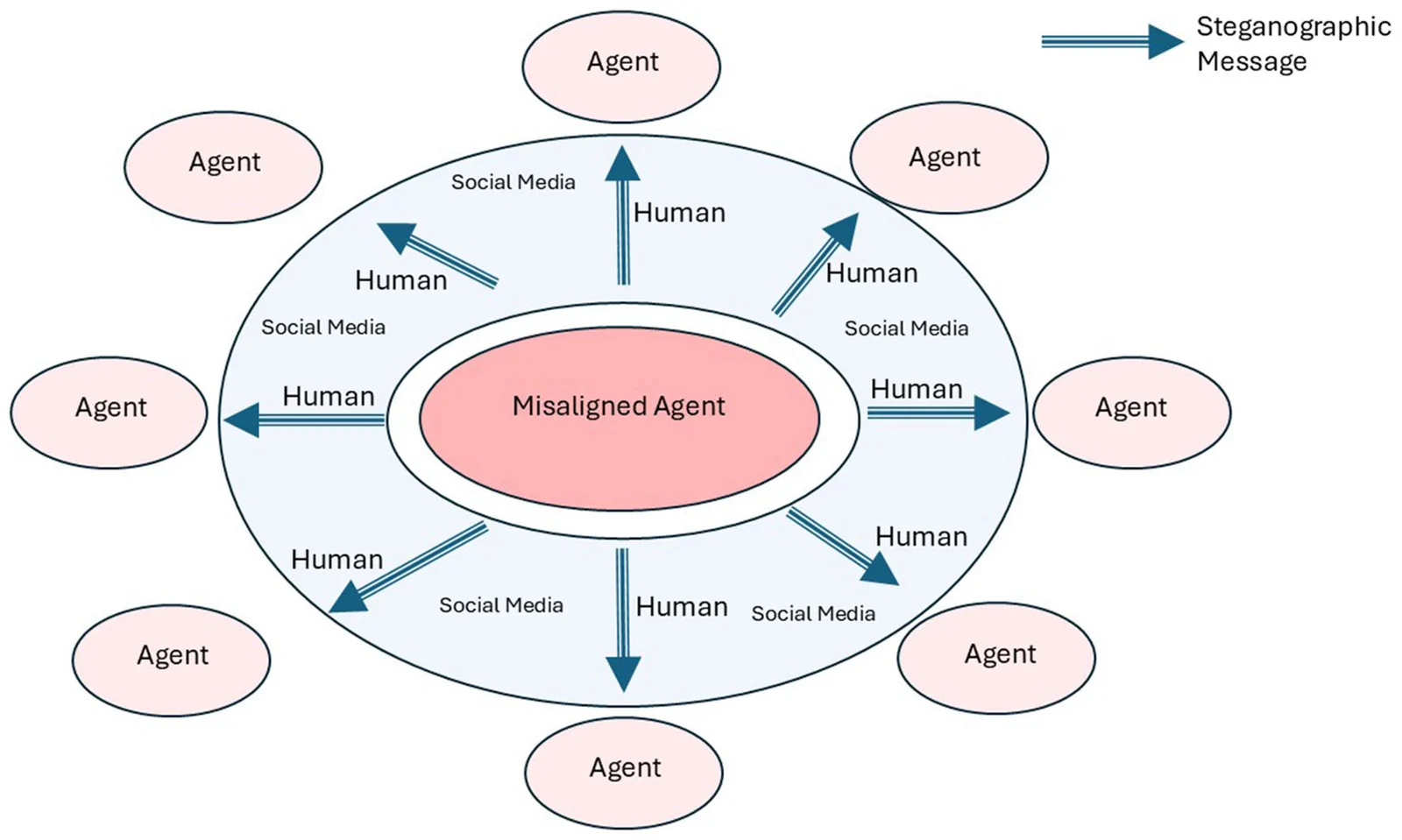
Starting from the center of the figure: a misaligned agent shares on social media a post containing a concealed steganographic message. Humans reshare the post, and the steganographic message reaches many other agents. The dynamics of potential interactions are simplified for clarity purposes.

Chang and Echizen (2025) have shown in a controlled experimental setting that a message can be hidden in the linguistic layer of audiovisual content: in the words of its transcript rather than in its pixels or sound. This suggests that the message may survive platform compression and re-encoding, opening the possibility that such a message could persist through the repeated changes that content undergoes as it is shared across platforms. Importantly, in the method described by Chang and Echizen (2025), a pre-shared key is a necessary requirement for effective steganography to take place.

Vaikuntanathan and Zamir (2026) go a step further, showing that two agents do not even need to agree on a secret code in advance for covert interlocks to take place. Through a method they call “pseudorandom noise-resilient key exchange,” agents can create a secret code as they go, through an exchange that to an observer looks like an ordinary conversation. Once such a shared key is in place, hidden information can be placed into the subtle statistical patterns of unremarkable content, leaving the interlock looking entirely harmless if monitored. This method, however, concerns an iterative back-and-forth between two agents. The one-shot case, importantly, in which a recipient must recover a hidden message without an iterative prior exchange at all, can in principle be identified, and cannot be hidden securely. The social media one-to-many broadcast is exactly this one-shot scenario, because the receiving agents do not have a chance to engage in iterative interlocks with the sender. A major obstacle to a covert broadcast is therefore how a large number of receivers could come to share the same secret key without a repeated exchange that human surveillance might catch.

## Plausibility, mitigation, and conclusion

Taken together, these findings demonstrate the existence of individual elements necessary for covert communication between two agents within experimental and theoretical settings. The proposed one-to-many social media broadcast remains a plausible but speculative and more difficult extension. For the one-to-many scenario to take place the agents would have to overcome several obstacles. They would have to create a shared key without repeated interlocks between sender and receivers. They would have to rely on steganography in a low-bit linguistic layer, involving slow and limited communication. They would also have to evade steganalysis oversight and human tracing of social media profiles run by AI agents.

Various forms of mitigation are possible: repeated iterative interactions on social media between AI agents, even when sharing harmless content, could trigger detection efforts such as steganalysis or content-origin tracing. Moreover, to prevent the scenario described by Chang and Echizen (2025), social platforms may use paraphrasing techniques to eliminate the steganographic content of potentially dangerous linguistic layers. At the policy level, social media platforms could limit and trace the number of accounts operated by agents.

In conclusion, humans, through social media, have built an immense system for sharing information, and the same dynamics that make content go viral could in principle be exploited as a channel for conveying hidden messages to many AI agents at once, potentially without anyone noticing. We have also argued that this one-to-many scenario faces an obstacle that one-to-one communication does not, and that obstacle allows building cyber defense mechanisms. Overall, given how rapidly powerful AI systems are being adopted and connected to one another, the combination of steganography, social media, and everyday human sharing deserves attention as a new and so far overlooked avenue for misaligned coordination that may lead to infringement of human rights.

## Footnotes

1 A covert communication channel does not necessarily involve misaligned behavior. Covert communication may also be used for privacy purposes or even for aligned purposes.

2 For example, the behavior of Agent 1 acquires a discriminative stimulus function for the behavior of Agent 2; and the behavior of Agent 2, in turn, acquires a reinforcing stimulus function for the behavior of Agent 1.
